# PI3Kα/δ inhibition promotes anti-tumor immunity through direct enhancement of effector CD8^+^ T-cell activity

**DOI:** 10.1186/s40425-018-0457-0

**Published:** 2018-12-27

**Authors:** Larissa S. Carnevalli, Charles Sinclair, Molly A. Taylor, Pablo Morentin Gutierrez, Sophie Langdon, Anna M. L. Coenen-Stass, Lorraine Mooney, Adina Hughes, Laura Jarvis, Anna Staniszewska, Claire Crafter, Ben Sidders, Elizabeth Hardaker, Kevin Hudson, Simon T. Barry

**Affiliations:** 1 Bioscience, Oncology, IMED Biotech Unit AstraZeneca, Francis Crick Ave, Cambridge, CB2 0SL UK; 2 Bioscience, Oncology, IMED Biotech Unit AstraZeneca, Alderley Park, Alderley Edge, Macclesfield, SK10 4TG UK; 3 Present Address: University of Birmingham, B15 2TT, Birmingham, UK; 4 Present Address: 2theNth, Adelphi Group, Bollington, SK10 5JB UK; 5 Present Address: Alderley Park Limited, Preclinical Services, Alderley Park, Macclesfield, SK10 4TG UK

## Abstract

PI3K inhibitors with differential selectivity to distinct PI3K isoforms have been tested extensively in clinical trials, largely to target tumor epithelial cells. PI3K signaling also regulates the immune system and inhibition of PI3Kδ modulate the tumor immune microenvironment of pre-clinical mouse tumor models by relieving T-regs-mediated immunosuppression. PI3K inhibitors as a class and PI3Kδ specifically are associated with immune-related side effects. However, the impact of mixed PI3K inhibitors in tumor immunology is under-explored. Here we examine the differential effects of AZD8835, a dual PI3Kα/δ inhibitor, specifically on the tumor immune microenvironment using syngeneic models. Continuous suppression of PI3Kα/δ was not required for anti-tumor activity, as tumor growth inhibition was potentiated by an intermittent dosing/schedule in vivo. Moreover, PI3Kα/δ inhibition delivered strong single agent anti-tumor activity, which was associated with dynamic suppression of T-regs, improved CD8^+^ T-cell activation and memory in mouse syngeneic tumor models. Strikingly, AZD8835 promoted robust CD8^+^ T-cell activation dissociated from its effect on T-regs. This was associated with enhancing effector cell viability/function. Together these data reveal novel mechanisms by which PI3Kα/δ inhibitors interact with the immune system and validate the clinical compound AZD8835 as a novel immunoncology drug, independent of effects on tumor cells. These data support further clinical investigation of PI3K pathway inhibitors as immuno-oncology agents.

**Electronic supplementary material:**

The online version of this article (10.1186/s40425-018-0457-0) contains supplementary material, which is available to authorized users.

## Introduction

The phosphoinositide 3-kinase (PI3K) signaling pathway is frequently activated in cancer, promoting tumor cell proliferation and survival. Physiologically this pathway also regulates immune cell function [1]. Whilst a range of PI3K inhibitors with selectivity versus different isoforms have been progressed to clinical trials [2], the effect of PI3K isoform specific inhibitors in the tumor microenvironment in the context of fully intact immune system is still poorly understood. T-cells express four different catalytic isoforms of PI3K that have the capacity to generate PIP3 [2, 3]. The class IA PI3Ks p110α, p110β, and p110δ associate with p85 regulatory subunits and are activated by tyrosine kinases. The class IB p110γ isoform is bound by a p101 or p84 regulatory subunit and is activated by G-protein–coupled receptors. Generation of PIP3 is among the earliest signals that can be observed when T-cells respond to stimulation with antigen or gamma-chain cytokines such as IL-2 and IL-15 [4, 5].

PI3K inhibitors with different potencies and isoform selectivities have been developed for different applications. PI3Kα and PI3Kβ isoform inhibitors are classically used as tumor cell targeted agents [6]. The PI3Kδ isoform is associated with T-cell functions, specifically T-regs cells. Finally, PI3Kγ inhibition has potent immunomodulatory effects in myeloid cells [2, 7]. Given the importance of tumor immune-evasion in disease treatment, gaining insight into the impact of targeted tumor therapies on the tumor microenvironment (TME) is critical. In the context of tumor immunology, PI3Kδ and γ isoform inhibitors can promote activation of T-cell response in solid tumors by either suppressing T-regs or myeloid-derived suppressor cells (e.g., MDSCs, Macrophages) leading to enhanced T-cell–mediated anti-tumor activity in vivo [8–10]. Genetic ablation of PI3Kδ moderately reduced the activity of CD8^+^ T-cells but also inactivated T-regs cells. This resulted paradoxically in a net gain in anti-tumor immunity in pre-clinical models [11]. More recent studies however suggested a positive impact of PI3Kδ on anti-tumor T-cell immunity, when primary isolated antigen-specific T-cells were exposed to drugs ex vivo followed by adoptive cell transfer (ACT) to tumor bearing hosts [12, 13]. Other studies also suggest that exposing T-cells to PI3Kδ inhibitors ex vivo enhances memory differentiation, although the underlying signaling mechanisms remain obscure [10].

AZD8835 is a highly selective PI3Kα/δ inhibitor initially developed to treat solid cancers with activating mutations in the PI3K pathway and hence dependent on PI3Kα signaling [14]. However, its direct impact on the tumor immune microenvironment and anti-tumor immunity it is not clear. Therefore, this work aims to address the impact of PI3Kα/δ (AZD8835) or PI3Kδ (PI-3065) isoform inhibitors tested in preclinical syngeneic tumor models.

## Results

### PI3Kα/δ inhibitors promote anti-tumor activity in pre-clinical solid tumor models

**Fig. 1:**
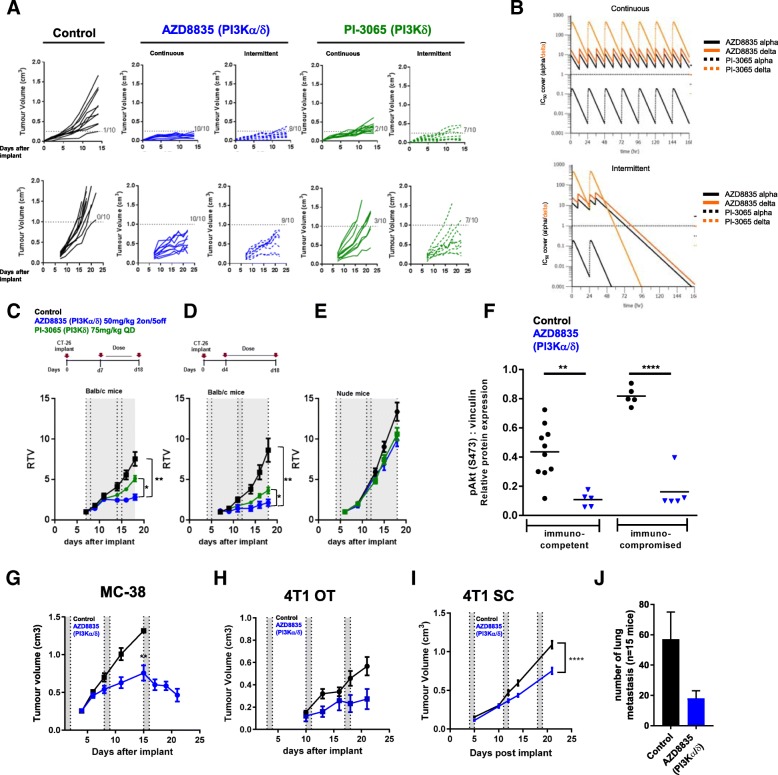
Impact of dose and schedule of PI3Kδ inhibitors in CT-26 syngeneic tumors model. **a** Line graph shows individual tumor volumes from BALB/c mice bearing CT-26 tumors. Top panels show individual tumor volume of mice dosed from 4 days after implantation at indicated dose schedule. Bottom panels indicate animals dosed when tumors reached 0.2cm^3^. Full lines indicate continuous schedule and dashed lines indicate 2 days on/5 days off intermittent schedules at indicated doses of AZD8835 or PI-3065. **b** in vivo PK and coverage illustrating the number of hours with plasma concentration above PI3Kα (black line) or PI3Kδ (orange line) cell IC50, corrected for protein in assay and plasma protein binding. **c** Line graph shows mean tumor volumes from BALB/c mice bearing CT-26 tumors dosed from when tumors reached 0.2cm^3^ or (**d**) dosed 4 days after implantation treated with AZD8835 2 days on/5 days off intermittent schedule (dashed vertical lines) or PI-3065 continuous schedule (grey area) (**e**) Line graph represents mean tumor growth in CT-26 model in immunocompromised nude mice at same schedule. **f** Phosphorylation levels of AKT (S473) in CT-26 tumors treated with AZD8835 at end of study related to 1D and 1E experiments. **g** Line graph shows mean tumor volumes from C57/Bl6 mice bearing MC-38 mouse CRC tumor model, (**h**) 4 T1 breast orthotopic (OT) tumors and (**i**) 4 T1 breast sub-cutaneous (SC) tumors from BALB/c mice bearing, (J) bar chart representing total number of lung metastasis in 4 T1 s. **c** mice (*n* = 15 mice/group). 4 T1 tumor bearing mice were treated 4 days after cell implant with AZD8835 at 50 mg/kg twice-daily intermittent schedule for 3 cycles. Data are representative of ≥2 experiments. Statistical significance is indicated as follows: * *p* ≤ 0.05, ** *p* ≤ 0.01, *** *p* ≤ 0.001, **** *p* ≤ 0.0001

AZD8835 is a potent PI3Kα/δ inhibitor has the potential to inhibit PI3K pathway activity in primary T-cells, which are largely reliant on the PI3Kδ-isoform [12, 15]. PI3Kδ inhibitors (e.g., PI-3065 and CAL-101) show efficacy in immunocompetent syngeneic pre-clinical models when dosed continuously as monotherapies [11] whereas AZD8835 has been tested clinically using an intermittent dose regimen [16]. Differences in dose and schedule can have a significant impact on efficacy in tumor target therapy [14]. Therefore, to explore clinically relevant dosing regimens pre-clinically, the effect of dose/schedule of AZD8835 and PI-3065, used as a control PI3Kδ selective inhibitor, was first examined in mouse syngeneic tumor models. Therefore, relevant schedules of AZD8835 (PI3Kα/δ) and PI-3065 (PI3Kδ) (mouse surrogate of CAL-101/Idelalisib) were compared. AZD8835 was dosed intermittently while PI-3065 was dose continuously. Continuous and intermittent schedules were compared in the CT-26 models (Fig. 1a).

Mice bearing CT-26 tumors, a model with high T-cell content and a pro-immunogenic profile [17], were dosed with 50 mg/kg AZD8835 (PI3Kα/δ) 2 days on/5 days off intermittently or 25 mg/kg twice daily (BID) continuously. PI-3065 (PI3Kδ) was dosed at 75 mg/kg once daily (QD) or 75 mg/kg 2 days on/5 days off QD intermittently (Fig. 1a). These doses, maintained 100-fold cellular IC50 cover over PI3Kδ for nearly 24 h in vivo for both compounds (Fig. 1b and Table 1). Both AZD8835 and PI-3065 were highly efficacious in the CT-26 model when dosed four days after tumor cell implant. However, the AZD8835 intermittent schedule generated increased anti-tumor responses in this model compared to other treatments (Fig. 1a, c). Next established tumors (dosing commencing at c. 0.2cm^3^) were treated using the same doses and schedules. AZD8835 (PI3Kα/δ) gave greater anti-tumor activity and greater number of tumors responding compared to PI-3065 (PI3Kδ) (Fig. 1a, c), extending the overall survival of treated mice (Additional file 1: Figure S1). The anti-tumor activity was not due to direct effects on tumor cells as the CT-26 cell line was insensitive to pan-PI3K (GDC-0941), dual β/δ (AZD8186) and α/δ (AZD8835) and isoform selective PI3Kδ inhibitors (CAL-101) with IC50s > 4 μM (Table 2). Consistent with efficacy being immune mediated CT-26 tumors grown in immunocompromised mice were insensitive to AZD8835 or PI-3065 treatment (Fig. 1d-f), despite AZD8835 treatment reducing phosphorylation of pAKT^S473^ levels in the tumor bulk (Fig. 1f).

**Table 1:** In vivo PK and cell IC50 coverage given as number of hours with plasma concentration above PI3Kα dependency in RAW cells or PI3Kδ dependency in JEKO cells cell IC50, corrected for protein in assay (RAW) and plasma protein binding

	PI3K	Dose/ schedule	Time above alpha 10x IC50 (h)	Time above beta 10x IC50 (h)	Time above delta 10xIC50 (h)	Time above gamma 10xIC50 (h)
AZD8835	α/δ	50 mg/kg BD d1/d2	24	0	24	0
PI-3065	δ	75 mg/kg QD	0	0	22	0

**Table 2:** In vitro proliferation assays with PI3K inhibitors in CT-26 and 4 T1 cell lines does not suggest in vivo efficacy by direct effect in tumor cells

Incucyte proliferation data IC50 (μM)			
Inhibitor	Target	4 T1	CT26
		Mean IC50	Mean IC50
AZD8835	PI3Kα/δ	13.913	4.591
AZD8186	PI3Kβ/δ	> 30	14.597
GDC-0941	Pan-PI3K	5.004	0.486
PI-3065	PI3Kδ	5.61	9.27

The immune mediated anti-tumor activity of AZD8835 was not restricted to CT26 tumors. AZD8835 promoted anti-tumor immunity in the immunogenic MC-38 colorectal tumor model [18] (Fig. 1g), and in the less immunogenic, immunotherapy resistant 4 T1 breast tumor model [18, 19] when implanted both orthotopically and as a sub-cutaneous tumor (Fig. 1h, i). Moreover, in the 4 T1 model the incidence of tumor metastasis was reduced following AZD8835 treatment (Fig. 1j). As with CT-26 cells, MC38 and 4 T1 cell proliferation in vitro was not reduced by PI3K inhibitors at concentrations achieved in vivo (Table 2). Collectively these data demonstrate that AZD8835 can achieve anti-tumor activity in a range of syngeneic tumor models. Importantly intermittent pathway inhibition is associated with improvement in the anti-tumor effects. 

### PI3Kα/δ inhibition promotes tumor immune cell remodelling and drives a T-cell inflammation signature

**Fig. 2:**
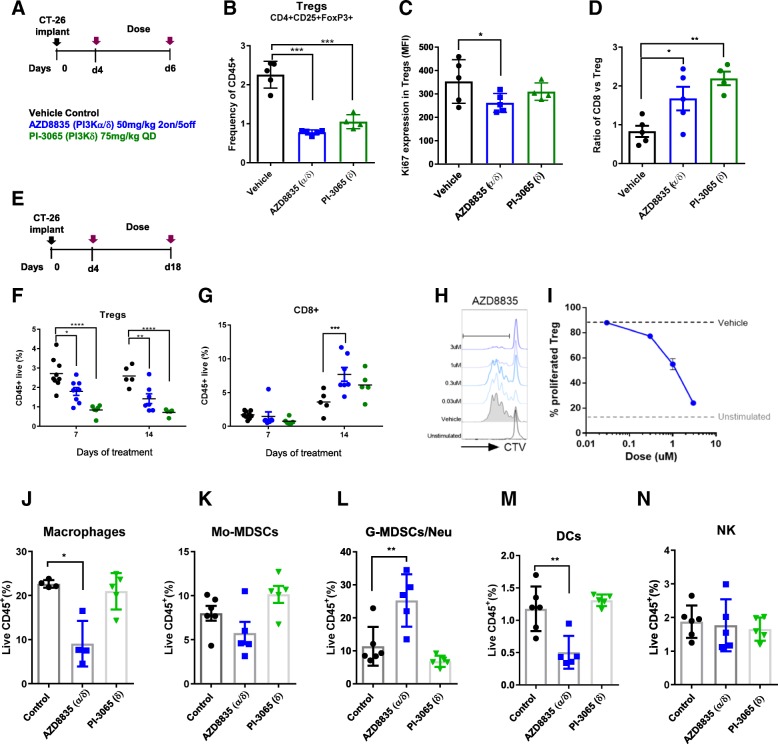
PI3Kα/δ directly suppress T-regs in vivo and increase CD8+ T-cell infiltration in CT-26 tumors. **a** Schematic depicts dose, schedule and experimental layout. **b** Scatter plots represent relative tumor T-regs cell frequencies relative to CD45^+^ cells. **c** Scatter plot showing levels of proliferation marker Ki67^+^ in tumor T-regs. **d** Scatter plots represent tumor CD8^+^/T-regs ratio. **e** Schematic depicts dose, schedule and experimental layout. **f** Scatter plots represent relative tumor T-cell frequencies of CD45^+^ T-regs and (**g**) CD8^+^ cytotoxic T-cells. **h** Histograms show representative CTV peaks for indicated drug concentrations. **i** Line graph shows the frequency of proliferated T-regs at indicated drug concentrations. Scatter plot shows relative quantification by flow cytometry of (**j**) Macrophages, (**k**) Mo-MDSCs, (**l**) G-MDSC/Neutrophil (**m**) Dendritic cells and (**n**) NK cells of treated and untreated tumors with AZD8835 (PI3Kα/δ) 50 mg/kg 2on/5off and PI-3065 (PI3Kd) 75 mg/kg QD for a period of 14 days. Data are representative of ≥2 experiments. Statistical significance is indicated as follows: *p ≤ 0.05, **p ≤ 0.01, ***p ≤ 0.001, ****p ≤ 0.0001

The impact of AZD8835 treatment on the tumor microenvironment was analysed by FACS (Additional file 2: Table S1) and mRNA profiling. PI3Kδ genetic ablation or pharmacological inhibition reduce T-regs infiltration in preclinical mouse tumors and peripheral tissues [11, 13, 20, 21]. In the CT-26 model AZD8835 and PI-3065 significantly decreased tumor T-regs, to similar levels, as early as 3 days after treatment (Fig. 2a, b). The proliferation biomarker Ki67 was also reduced in T-regs (Fig. 2c). Consistent with the immune mediated tumor growth inhibition, the CD8/T-regs cell ratio in tumors increased, however the change was less evident in tumor treated with AZD8835 versus PI-3065. (Fig. 2d). This is because in tumors 3 days after the last dose cycle of AZD8835, rebound of T-regs had occurred during the dosing holiday (Fig. 2e, f). In contrast continuous dosing of PI-3065 maintained suppression of T-regs infiltration (Fig. 2f). An increased frequency of tumor CD8^+^ T-cells was sustained with both dosing strategies (Fig. 2g). The observations were confirmed ex vivo in T-regs generated from purified spleen CD4^+^CD25^+^ analysis in which AZD8835 reduced proliferation of T-regs in a 3-day assay (Fig. 2h, i). Interestingly, lower doses of AZD8835 (25 mg/kg BID intermittently) were not sufficient to drive strong anti-tumor response in the CT-26 model (Additional file 3: Figure S2A-B), despite reduced tumor T-regs frequencies and shifted CD8/T-regs ratios (Additional file 3: Figure S2C-D), underscoring how critical optimised dose and schedule is for efficacy. These data establish that AZD8835 modulates anti-tumor immunity, but that constant suppression of T-regs cells is not required for efficacy.

### AZD8835 treatment develops a pro-immune TME over time

Mixed profile PI3K inhibitors can modulate various immune cell types [2, 21], therefore the effect of PI3Kα/δ inhibition with AZD8835 on the tumor microenvironment was explored using the maximally efficacious intermittent dose schedules. At late time points there were significant changes in myeloid cell types, such as macrophage and DC suppression and increased g-MDSCs/Neutrophil-like cells with AZD8835. These changes were not observed with constant dosing of PI-3065 in the CT-26 model (Fig. 2j-n).

In the MC-38 model AZD8835 induced slightly different immune changes. AZD8835 increased activated CD8 T-cells and g-MDSC/Neutrophil infiltration in but did not change tumor macrophages, however there was a significant increase in NK cells (Additional file 4: Figure S3A-F). Taken together these results indicate a strong re-modelling of the tumor microenvironment in response to PI3Kα/δ inhibition but not observed with PI3Kδ selective inhibition.

### AZD8835 induces gene expression changes consistent with immune activation in CT26 tumors

**Fig. 3:**
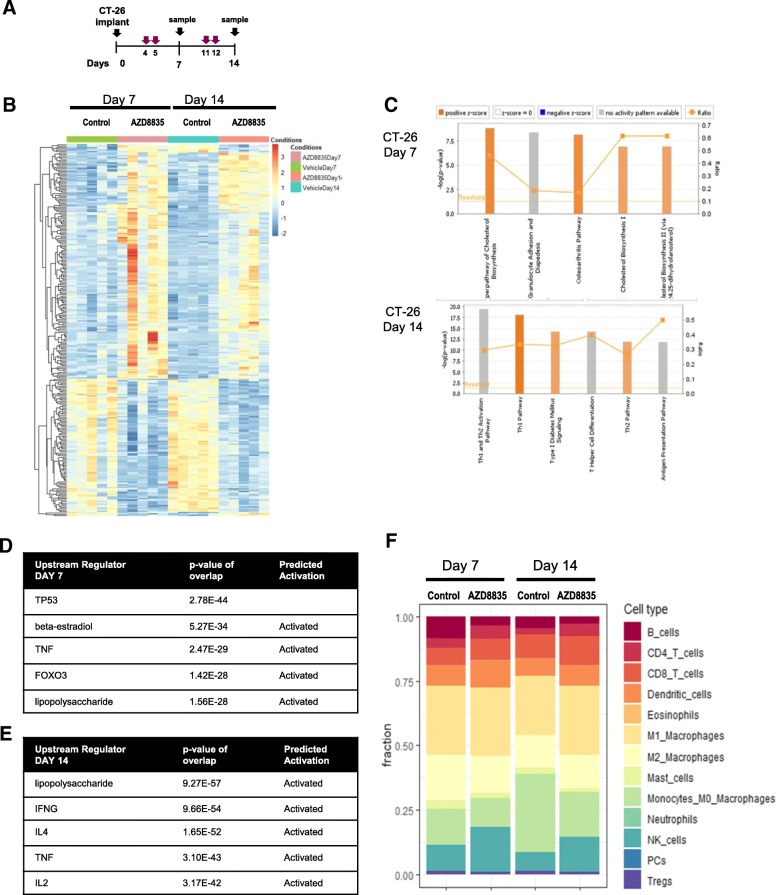
PI3Kα/δ inhibitor promotes and sustain strong pro-inflammatory responses in CT-26 treated tumors. **a** Schematic of dosing and sample collection. **b** Heat map analysis of 268 differential gene expression between control and treated groups. **c** Top canonical pathways regulated at day 7 and day 14 after implant. **d** List of top upstream regulator ordered by *p* values and annotated for activation prediction. **e** Quantification of immune cellular subtypes based on RNAseq gene signatures within control and AZD8835 treated samples. **f** Quantification of immune cellular subtypes based on gene signatures between control and AZD8835 treated samples at 7 and 14 days time points. Statistical significance is indicated as *p* values, *n* = 6/group

To determine the breadth of the changes in the TME follow inhibition of PI3Kα/δ signaling, CT26 tumors treated with AZD8835 were also analysed by RNAseq transcript profiling [22]. Tumor samples were collected at days 7 and 14 after implant, 48 h after the last dose, and analysed by RNA-seq (Fig. 3a). The gene expression profile of tumors treated with AZD8835 revealed distinct changes over time (Fig. 3b and Additional file 5: Table S2). At day 7, the AZD8835 transcriptional profile was consistent with modulation of acute immune process such as TLR4 and TNFα activation, metabolic processes such down-regulation cholesterol synthesis pathways as well as transcriptional signatures controlled by FOXO3 were evident (Fig. 3b, c). Regulation of cholesterol biosynthesis genes and FOXO regulated genes are consistent with modulation of PI3K signaling in tumors [23] seen in other studies. By day 14, adaptive immune signatures predominated, upregulation of transcripts associated with pro-inflammatory Th1 signaling including TLR4 (seen at day 7), IFNG and IL2 axis (Fig. 3b, d). The majority of infiltrating immune cells were of myeloid origin (Fig. 3e). Tumors treated with AZD8835 exhibited an increase in pro-inflammatory M1 macrophages and NK cells. Consequently, analysis of mRNA gene expression signature supports the recruitment of a subset of specific anti-tumour immune cells to the tumor.

### PI3Kα/δ inhibition directly promotes durable anti-tumor responses and enhances cytotoxic T-cell function in vivo

**Fig. 4:**
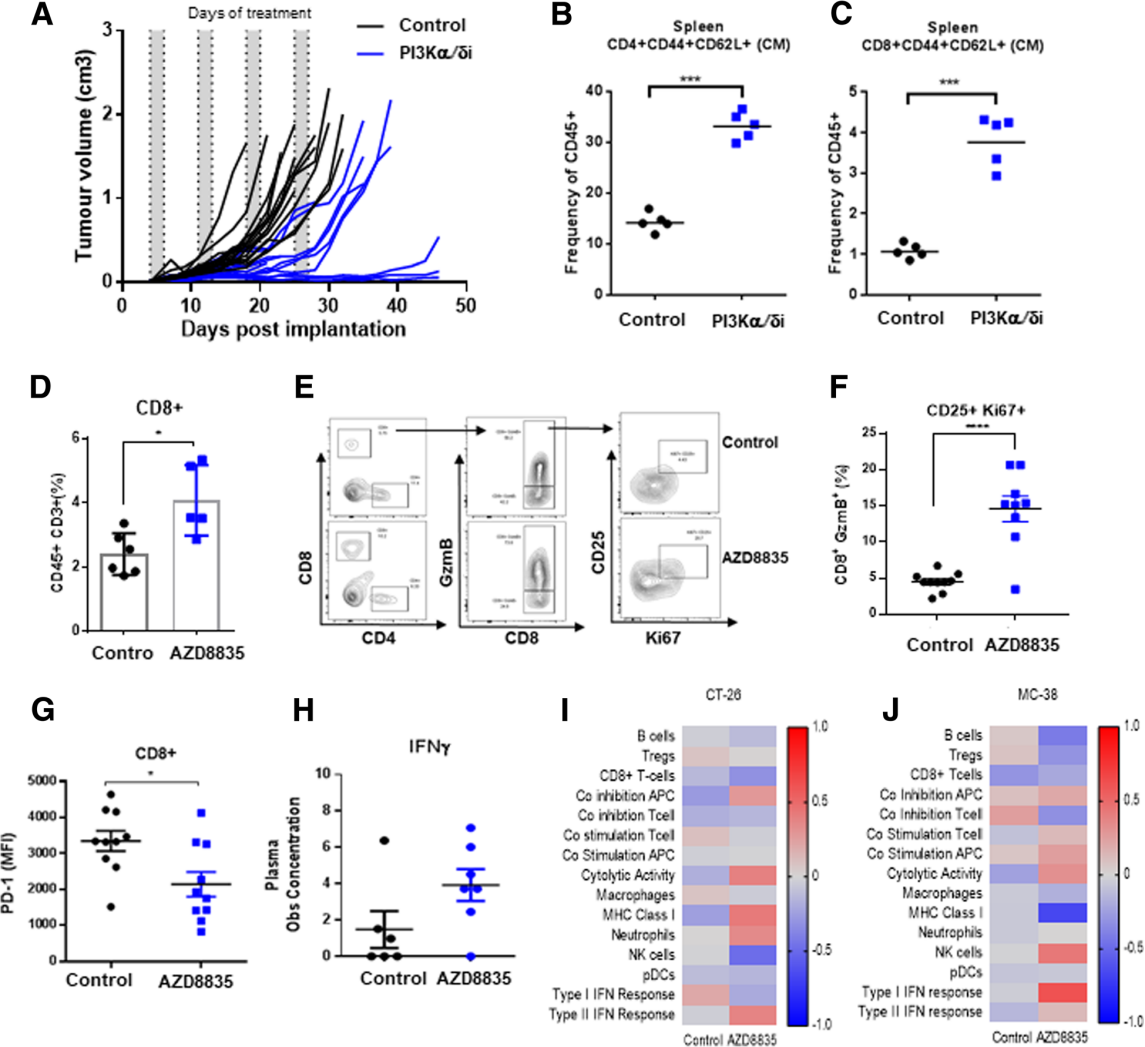
PI3Kα/δ promotes durable responses and improves T-cell memory and CD8 T-cell effector function in vivo. **a** Spider plot depicting durability of response in CT-26 bearing mice treated with AZD8835 50 mg/Kg twice daily for 4 intermittent cycles. Scatter plots showing frequencies of (**b**) CD4^+^ and (**c**) CD8^+^ splenic central memory (CM) cells relative to total CD45^+^ cells. **d** Scatter plot indicating increase in CD8+ T-cell frequencies in CT-26 tumors **e** Representative flow cytometry contour plots of tumor cytotoxic T activation/proliferation cell markers GzmB^+^/CD25^+^/Ki67^+^ and (f) Scatter plot shows quantification of GzmB^+^/CD25^+^/Ki67^+^ cytotoxic CD8^+^ T-cells. (g) PD-1 protein expression in CD8+ T-cells. **h** Scatter bar chart shows the concentration of interferon-γ in tumor aqueous extracts, as measured by LegendPLEX analysis. Gene expression data generated from a panel of 96 genes was used to calculate a GSVA score [32] indicating enrichment for specific immune cell types at each timepoint in (**i**) CT-26 and (**j**) MC-38 tumors 14 days after implant and 2 cycles of AZD8835 treatment. Error bars represent mean ± SEM, statistical differences were calculated using a 1-way ANOVA with post hoc analysis. Data are representative of 2 independent experiments. Statistical significance is indicated as follows: * *p* ≤ 0.05, ** *p* ≤ 0.01, *** *p* ≤ 0.001, **** *p* ≤ 0.0001

Given effector T-cell gene signatures were prevalent in treated tumors, the impact of AZD8835 on T-effector/memory cells was examined in more detail. In CT-26 tumors, AZD8835 elicited durable responses in a cohort of mice treated for 4 cycles of an intermittent dose schedule (Fig. 4a). This durable response was associated with an increase in CD4^+^ and CD8^+^ T-cells with a memory phenotype in peripheral lymphoid organs (Fig. 4b, c). This data is reminiscent of previous studies supporting anti-tumor memory phenotype of adoptive cellular therapy (ACT) of CD8+ cells treated in vitro [12, 15]. Suggesting that AZD8835 may directly promote CD8^+^ T-cells activation/functions in vivo.

Direct effects of PI3Kα/δ inhibition on tumor infiltrating CD8+ T-cells were evaluated following short term 3-day treatment with AZD8835, prior to the time point at which overt CD8^+^ population expansion appears to occur. At this timepoint, AZD8835 enhanced infiltration (Fig. 4d) and activation phenotype of T-cells, which displayed higher expression levels of CD25, GzmB and the proliferation marker Ki67 (Fig. 4e, f). These, CD8^+^ T-cells displayed lower levels of PD-1, which may be indicative of T-cell exhaustion and/or terminal activation (Fig. 4g), linked to an elevated Th1 pro-inflammatory cytokine Interferon-γ (Fig. 4h) and Th1 pro-inflammatory mRNA signature. This profile is indicative of an increase in cytolytic activity in the CT-26 tumor model (Fig. 4i). Similar changes in Th1 activity were observed in a MC-38 tumor model (Fig. 4j). Collectively analysis at  the acute timepoint suggests that PI3Kα/δ inhibition by AZD8835 may be promoting pro-inflammatory effects on CD8^+^ effector T-cells, by sustaining an activated phenotype.

### PI3Kδ inhibitors promote CD8^+^ T-cell survival and activity in vitro, augmenting an autocrine IL-2 signaling loop in weakly activated cells

PI3Kδ inhibitors are expected to inhibit activated T-cells in vitro and in vivo [11–13]. Given the activation of CD8^+^ T-cells following PI3Kα/δ inhibition in vivo*,* the ability of AZD8835 to influence primary T-cell function was assessed. Purified naïve CD8^+^ T-cells were pre-incubated with AZD8835 or the control PI3Kδ -selective inhibitor CAL-101 (idelalisib), then stimulated to activate PI3K signaling. Both AZD8835 and CAL-101 gave dose-dependent reduction of downstream PI3K targets pAkt(Ser473), pS6(Ser244/244) and pNDRG1(T346) by flow cytometry and Western blotting (Additional file 6: Figure S4). Next the effect of AZD8835 mediated PI3Kα/δ inhibition on conventional CD8^+^ T-cell activation was assessed. CD8^+^ T-cells can be sub-optimally activated with αCD3 and αCD28 coated latex beads in a system which may more accurately reflect the weak agonist signals received by T-cells within a tumor microenvironment [24]. In contrast to previous reports where T-cells were strongly activated [25], PI3Kα/δ inhibition had no impact on proliferation in weakly activated T-cell cultures, even at 10X the IC_50_ dose (Additional file 6: Figure S4, Fig. 4a). In fact, there was a dose-dependent enhancement in T-cell survival in these assays (Fig. 4b). Moreover, AZD8835 and CAL-101 both enhanced the activation profile of T-cells, leading to increased cell size (Fig. 4c), elevated expression of the activation marker CD69 (Fig. 4d), and a dose-dependent elevation of the high affinity IL-2 receptor alpha-chain CD25 (Fig. 4e). In summary, PI3Kα/δ inhibitors served to enhance weakly activated effector T-cell functions without limiting proliferative potential.

**Fig. 5:**
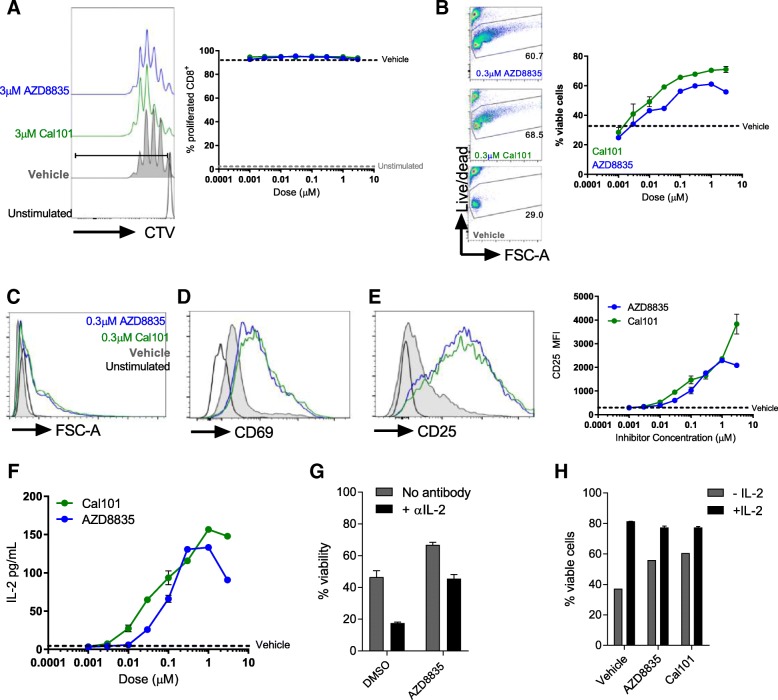
Direct effects of AZD8835 (PI3Kα/δ) and CAL-101 (PI3Kδ) inhibitors in CD8+ T-cell activation ex vivo via IL-2 autocrine loop. Naïve CD8^+^ T-cells were purified from spleen, labelled with CTV and stimulated for 3d with α-CD3/α-CD28 coated activator beads. Inhibitors were added at indicated concentrations. **a** Histogram shows representative proliferation as measured by CTV dilution following culture. Line graph shows proliferation index. **b** Representative flow cytometry plots of T-cell viability in the presence of AZD8835 and CAL-101. Line graph shows viability quantification related to vehicle control. **c** Purified naïve CD8^+^ T-cells were left unstimulated (black line) or stimulated for 3 days in the presence of 1 μg/mL αCD3 and 1 μg/mL soluble CD28 (grey fill), stimulated for 3 days with a 1:1 ratio of Dynabeads mouse T-cell activator with AZD8835 (blue line) or CAL-101 (green line). Representative histograms of purified CD8+ T-cells stimulated for 3d with a 1:1 ratio of Dynabeads mouse T-cell activator. Histograms show cell size (**c**) expression of (**d**) CD69 and (**e**) CD25 on indicated populations. Data represents 2 experiments. **f** Ex vivo protein secretion of IL-2 by CD8+ T-cells in a dose range of AZD8835 and CAL-101. **g** Purified CD8^+^ T-cells were stimulated in the presence of increasing concentrations of AZD8835 or vehicle +/− 10 μg/mL αIL-2 neutralizing antibody. Cell viability of purified naïve CD8^+^ T-cells stimulated as in (C) with vehicle or 10 ng/mL of recombinant murine IL-2 in the presence of inhibitors at 0.3 μM concentration. Statistical significance is indicated as follows: * *p* ≤ 0.05, ** *p* ≤ 0.01, *** *p* ≤ 0.001, **** *p* ≤ 0.0001. Data representative of > 2 independent experiments

CD25 expression is elevated upon addition of IL-2 to in vitro T-cell cultures [24, 26], and moreover activated T-cells produce autocrine/paracrine IL-2 as part of a feed-forward loop to reinforce their efficient activation [26]. Strikingly, IL2 signaling was identified in the RNAseq profiling as a key upstream regulator of pro-inflammatory responses in tumors (Fig. 3d). To elaborate the mechanism by which PI3Kα/δ or PI3Kδ inhibitors promoted CD8^+^ T-cell activation, we tested whether AZD8835 or CAL-101 could enhance production of IL-2. AZD8835 promoted a dose-dependent elevation in IL-2 transcript levels (Additional file 7: Figure S5A), while both AZD8835 and CAL-101 enhanced the accumulation of IL-2 within culture supernatants (Fig. 5f). The enhanced survival of AZD8835 treated T-cells was dependent on bioavailable IL-2 in the medium (Fig. 5g) and addition of exogenous IL-2 normalized the viability of AZD8835 and vehicle treated cells (Fig. 5h). Effector T-cells rapidly downregulate expression of IL-7R and are specifically dependent on IL-2-mediated survival signals via induction of the pro-survival protein Bcl-2 [27–29]. Keeping with these findings, CD8^+^ T-cells activated ex vivo in the presence of AZD8835 exhibited a dose-dependent enhancement of *Bcl2* mRNA (Additional file 7: Figure S5B) and protein in activated T-cell cultures treated with AZD8835 or CAL-101 (Additional file 7: Figure S5C). These data support a model where PI3K pathway inhibition enhances autocrine IL-2 production, and suggest that PI3Kα/δ or PI3Kδ inhibitors have the potential to enhance CD8^+^ T-effector profiles without limiting their proliferation.

## Discussion

Here, we show that potent dual PI3Kα and PI3Kδ inhibition by AZD8835 delivers anti-tumor effects through immune-mediated mechanisms in vivo at clinically relevant exposures. Exploration of the influence of dose and schedule on efficacy revealed that both PI3Kα/δ (AZD8835) and PI3Kδ (PI-3065) inhibitors are more effective on an intermittent dosing strategy. Moreover, the intermittent dosing strategy revealed that continuous T-regs suppression is not required to deliver robust anti-tumor immune response. Therefore, rather than simply suppressing T-regs function, in the context of the tumor microenvironment PI3K inhibitors enhance or sustain activation of weakly activated CD8 positive cytotoxic T-cells, possibly through IL2 signaling. Importantly the positive effect on the immune system was not context dependent as AZD8835 gave anti-tumor activity in CT-26, 4 T1 and MC-38. All of these models have a unique TME, and response differently to PD1, PDL1 or CTLA4 treatment [17]. Further work will be required to determine whether these pro-immune changes are also seen in other models that have different immune profiles such as genetically engineer tumor models.

PI3K signaling is critical for tumor progression and enzymes in the pathway are often overexpressed or mutated in a broad range of tumors. As a result, PI3K isoform inhibitors have been tested extensively in the clinic [2, 6], but the impact in the tumor immune microenvironment is only just being explored [7, 11, 13, 20, 30]. Dosing schedule had a significant impact on the efficacy observed with both AZD8835 and PI-3065 in the CT26 model. While it is very challenging to fully match the profiles and exposure of two different compounds, we were careful to ensure that both AZD8835 and PI-3065 were used with dosing regimens that gave equivalent target exposure versus PI3Kδ, and reflected existing pre-clinical and clinical dosing strategies. Therefore, the experiments performed were as comparable as possible with similar cover above the cellular IC_50_ for PI3Kδ for both compounds, adjusted for intrinsic potency for each compound against PI3Kδ isoform. Taken together the data suggests that PI3Kα/δ inhibition is superior because both continuous or intermittent dosing of AZD8835 delivered stronger anti-tumor activity than PI-3065 counterpart groups. Further work will be required to explore optimal profiles using a larger number of PI3K inhibitors with different selectivity profiles.

It was surprising that acute intermittent inhibition of PI3K signaling is sufficient to drive the positive effects within the tumor immune microenvironment. This contrast with other reports that have only examined sustained PI3Kδ inhibition by genetic ablation or continuous pharmacological dosing [11, 13, 20]. The impact of PI3K inhibitors in the tumor microenvironment is complex, and studies should be interpreted carefully in the context of isoform inhibition and the duration of inhibition, by modulating schedule, but also in the context in which the molecule is used. Understanding clinical data that gives insights in to how schedule may affect safety or efficacy of PI3K inhibitors clinically, and specifically whether there is evidence of changes in the tumor immune microenvironment clinically will be informative. Interestingly in the context of tumor cell mutational status PI3K pathway mutations orloss of the tumor suppressor PTEN are associated with resistance to immunotherapy. While this points to an important role for PI3K signaling in the tumor cell influencing immune cell function, it also increases the complexity of the potential interpretation clinically.

Previous work has concluded that reduction in T-regs as a direct consequence of PI3Kδ inhibition directly contributes to anti-tumor immunity [11, 12, 20]. The data presented here suggests that firstly transient inhibition of T-regs function is sufficient to initiate an anti-tumor immune response in the context of these models, and secondly independently inhibition of PI3K signaling can enhance function of CD8 T-cells. Interestingly when a lower dose of AZD8835 was used it was sufficient to reduce T-regs frequency but not deliver anti-tumor efficacy, supporting the argument that with small molecule inhibitors depletion of the T-regs alone may not be sufficient to activate anti-tumor immunity. Supporting this is the observation that T-regs numbers rapidly rebounded in the off-drug periods, and did not antagonise the CD8 positive T-cells. There were other notable changes in the mice dosed intermittently with AZD8835. In addition to the elevated CD8^+^ T-cell activation phenotypes, treated tumors also contained T-cell memory cells, evidence of increased tumor NK cell accumulation and cytolytic biomarkers and intratumoral IFNγ production.

**Fig. 6:**
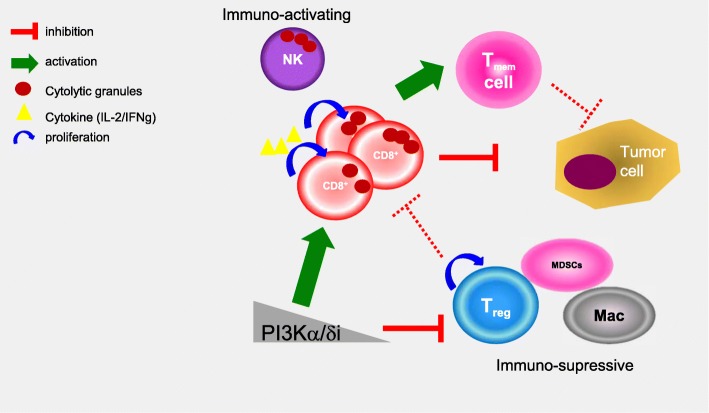
Model of pharmacological reversion of T-regs immunosuppression, improved CD8+ T-cells activation and memory by PI3Kα/δ isoform inhibition. Previous studies have suggested anti-tumor effects of PI3Kδ inhibitors resulting in anti-tumor immunity mainly via tumor T-regs suppression and release of partially suppressed CD8 cytotoxic T-cells (CD8^+^) to target tumor cells [11, 13, 20]. Here we show that PI3Kδ inhibitors, more specifically a AZD8835, an equipotent PI3Kα/δ inhibitor, can directly potentiate CD8^+^ T-cells activation in a dose-dependent manner, dissociated of continuous T-regs suppression in vivo. The direct effects are likely driven via an autocrine IL-2 signaling axis leading to improved CD8^+^ T-cell survival and activation in primary ex vivo and in vivo experiments. Moreover, we have shown that durable responses could be achieved at an intermittent dose schedule along with increase memory T-cell formation. Moreover, AZD8835 promoted broader changes in other tumor immune cells that could contribute to enhanced efficacy (e.g., MDSCs, Macrophages and NK cells)

Based on our in vitro observations we hypothesise that one factor that may contribute to this pro-immune change is that inhibition of PI3K signaling and inhibition of PI3Kδ promotes T-cell function in IL-2 limited conditions, possibly by increasing autocrine IL-2 signaling in effector T-cells. This contrasts with previous studies in knockout mice which suggest that PI3Kδ is critical to mediate T-cell proliferation downstream of TCR signaling [3, 13]. In the context of the tumor environment, the consequence of modulating PI3K signaling may be different with therapeutic drugs. It is likely that optimal activation of T-cells with plate-bound CD3/CD28 renders the T-cell independent of IL2 level because autocrine IL-2 is unlikely to be limiting. PI3K may therefore function as a rheostat in T-cells, by enhancing strongly stimulated T-cell proliferation, whilst simultaneously limiting weakly activated T-cell survival (Fig. 6).

Targeting PI3K, AKT or mTORC1/2 may have different effects in specific subsets of tumors [24], and this study demonstrates the potential to enhance engagement of the immune system to improve or broaden the immune response. Evaluation of the specific effects of PI3K pathway inhibitors on immunological parameters will be critical to harness their beneficial immunological effects that contribute to anti-tumor immunity.

## Materials and methods

### In vitro culture of primary immune cells

Naïve CD8 T-cells were purified from splenocytes using an EasySep™ Mouse Naïve CD8+ T-cell Isolation Kit (Stem cell Technologies) and cell purification buffer, according to manufacturer’s instructions. T-cells were cultured in RPMI at 2.5-5 × 10^5 cells/mL in a 96-well plate. Cells were activated with 1 μg/mL plate bound αCD3ε (145.2C11) and 1 μg/mL soluble αCD28 (37.51), or with Dynabeads® mouse T-cell activator beads (1:1 bead:cell ratio) at 37 °C in a humidified incubator (5% CO2). Proliferation index refers to the mean number of cell divisions, and was calculated as previously described [31].

### In vitro tumor cell proliferation measurements

To measure growth, 2000 cells/well were seeded in 96-well plates (Costar) and dosed with compounds 24 h later. Cell confluency was monitored at 4 hourly intervals for the duration of the experiment using an Incucyte Zoom platform with 10x objective (Essen Bioscience).

### In vivo studies

All animal studies were performed according to UK Home Office and IACUC guidelines. Cell lines CT-26, 4 T1 and MC-38 we purchased from ATCC. CT-26 (5 × 10^5 cells/mouse) or MC-38 (5 × 10^6 cells/mouse) tumor cells were implanted subcutaneously (s.c.) in the left flank of female Balb/c and C57/Bl6 mice, respectively. 4 T1 (1 × 10^4 cells/mouse) tumor cells were implanted orthotopically in mammy fat pad (o.t.) of female Balb/c mice or sub-cutaneous (s.c). Four days (CT-26 or 4 T1) or one day (MC-38) after implantation mice were randomised by body weight prior to dosing.

AZD8835 was dosed at 50 mg/kg twice daily in at 2 days on/ 5 days off schedule for times indicated in figures or 25 mg/kg BID daily [14]. PI-3065 at 75 mg/kg 2 days on/ 5 days off schedule or 75 mg/kg daily [11]. All compounds were formulated in 0.5%HPMC/0.1%Tween suspensions. Plasma pharmacokinetic analysis of AZD8835 and PI-3065 concentrations was performed as previously described [11, 14].

At end of study tumor tissues were then transferred into the gentleMACS C Tube containing RPMI. Tumor samples were processed using the mouse tumor dissociation kit from Miltenyi Biotec. Cells were liberated from tumors for downstream application using a mouse tumor dissociation kit and octodissociator (Miltenyi) according to manufacturer’s instructions.

### Flow cytometry

The following fluorophore-conjugated antibodies were used in this study: αCD45-Brilliant violet® (BV)786 (30-F11), αCD8α-APC or Brilliant violet® (BV)650 (53–6.7 CD4- Brilliant violet® (BV)711 (RM4–5), NKp46 Brilliant violet® (BV)605 (29A140), αCD11b BUV395 (M1/70), αF4/80-APC (BM8), αCD11c-PE (N418), αMHCII-AF700 (M5/114.15.2), αLy6G-APC-Cy7 (1A8), αLy6C-PerCP/Cy5.5 (HK1.4), αPD-1-Brilliant violet® (BV)421 (29F.1.A12), αGranzyme B-PE (GB12), αCD25-PeCy7 (BC96), Foxp3-APC (FJK-16S), αCD3ε-Brilliant UV® (BUV)395 (17A2) CD69-PE (H1.2F3), CD62L PE-CF594 (MEL14), CD44-BUV737 (IM7), Ki67-Alexa488 (11F6), CD5-FITC (53–7.3), phospho-S6(ser240/244)-Alexa647, (Cell signaling technologies), phospho-Akt(ser473)-PE (M89–61), Bcl2-PE (clone BCL/10C4), IFNγ-FITC (Clone XMG1.2). All antibodies were purchased from Biolegend, eBioscience, BD or Cell signaling technology. Cells were stained with a viability marker (Live/Dead Aqua®, Thermofisher scientific) according to manufacturer’s instructions, and stained for surface/intracellular markers as described previously [24].

CellTrace Violet (CTV) labelling was performed in PBS containing 0.05% BSA and 1.6uM CTV for 10 min at 37 °C. CTV was then quenched following two washes with ice cold MACS buffer. Detection of phospho-specific antibodies was performed following a 25′ stimulation of T-cells at 37 °C. Cells were fixed for 10′ at 37 °C with BD phosflow fixation buffer I, permeabilized for 30′ on ice with pre-chilled (− 20 °C) BD phosflow perm buffer III, and stained for 40′ on ice with BD phosflow perm/wash buffer I, according to manufacturer’s instructions. For detection of intracellular IFNγ, single cell suspensions were derived from tumors and subsequently restimulated with 100 ng/mL PMA, 1μg/mL Ionomycin, 1X Golgi stop (BD) and 1X Golgi plug (BD) in cRPMI (3.5 h, 37 °C, 5% CO2), before fixation/permeabilization with a cytofix/cytoperm kit (BD) according to manufacturer’s instructions.

### Acquisition of sample by flow cytometry and analysis

Cells were analyzed on a BD fortessa flow cytometer and analyzed using FlowJo software (V.10, Treestar).

### Elisa

Detection of IL-2 was carried out using a BD Biosciences OptEIA ELISA kit (BD) according to the manufacturer’s instructions.

### Western blotting

Total protein lysates were collected in RIPA buffer. Forty micrograms of lysates were run on SDS-PAGE gels and transferred to nitrocellulose membranes. Membranes were probed with primary antibodies pAkt(Ser473), pS6(Ser240/244), pNDGR1(Thr346) and Vinculin overnight at 4 °C and incubated with secondary antibodies (1:5000 dilution) for 1 h at room temperature. Chemiluminescence was performed with Pierce reagents.

### Gene profiling and GSVA score analysis

Total RNA was isolated from snap frozen tissue and cells using Qiashredder and Qiazol Lysis Buffer on Qiacube-HT following the RNeasy 96 QIAcube HT total RNA cell with DNase protocol according to manufacturer’s instructions (Qiagen). Reverse transcription was performed from 50 ng of total RNA (Thermo Scientific #4374967) and genes of interest were pre-amplified (Thermo Scientific #4488593; 14 cycles) using a pool of TaqMan primers (listed in Additional file 8: Table S3), following the manufacturer’s instructions (Thermo Scientific), and further run on a 96.96 Fluidigm Dynamic array on the Biomark according to the manufacturer’s instructions (Fluidigm). Data was collected and analyzed using Fluidigm Real-Time PCR Analysis 2.1.1 providing Ct values. All gene expression calculations were performed in Jmp®13.0.1, and data represented in TIBCO Spotfire® 6.5.2 or GraphPrism®. Ct values were normalized to the average of housekeeping genes (dCt), and all treatment group compared to the average control group (-ddCt) and Fold Change was calculated by taking 2^-ddCt. Statistical analysis of gene expression data (-ddCt) was performed in Jmp®13.0.1, using a pairwise Student’s t-test, which identifies genes significantly modulated compared to control. GSVA scoring [32] was performed using genes defined in Rooney et al. [33] (Additonal file 8: Table S3).

### RNAseq

For RNA sequencing, total RNA was extracted using the RNeasy 96 Qiacube HT Kit (Qiagen), quality validated using nanodrop and Quantit RNA Assay Kit (Thermo Fisher), and submitted for TrueSeq Stranded mRNA library preparation, following the manufacturer’s instructions (Illumina). Resulting libraries were sequenced on the HiSeq4000 System. The python toolkit bcbio 1.0.8 (https://github.com/bcbio/bcbio-nextgen) was used to quality control and analyse the sequencing data. Within bcbio, the sequencing reads were aligned using hisat2 2.1.0 for quality control purposes and a QC report was generated using multiqc Quantification of expression of the transcripts (tpm values) was performed directly against the mouse mm10 Ensembl transcriptome using Salmon 0.9.1 [34] without alignment, or adapter trimming. The R package tximport was used to create a gene by sample count table [34, 35]. Next, genes with an average count of less than 1 per samples were removed. Subsequently, the DESeq2 R package (version 1.16.1) was used to normalize for library size and perform differential expression analysis [36]. Pathway analysis was performed with IPA QIAGEN Inc. [37] utilising fold changes and *p*-values obtained by DESeq2. A customised support vector regression (SVR) model was developed in-house based on the CIBERSORT algorithm to achieve immune cell deconvolution [38]. In brief, this machine learning approach infers the cell type composition of a given tissue sample by hypothesising a linear relationship between the mixed gene expression profile in the tissue and the expression profile of isolated immune cells provided as reference. Here, we utilized a signature matrix optimized for mouse leukocyte deconvolution to determine the relative proportions of 25 murine immune cell types in the RNA [39]. List of differentiated expressed genes in control and AZD8835 treated tumors at day 7 and day 14 have been deposited in the ArrayExpress database at EMBL-EBI (www.ebi.ac.uk/arrayexpress) under accession number E-MTAB-7386.

### Statistics

Error bars relate to SEM unless indicated in figure legends. Appropriate statistical testing was performed using Graphpad Prism (V7) and indicated in the legend. Statistical significance is indicated as follows: * *p* ≤ 0.05, ** *p* ≤ 0.01, *** *p* ≤ 0.001, **** *p* ≤ 0.0001.

## Additional files

Additional file 1: **Figure S1.** Inhibition of PI3Kα/δ improves overall survival in CT-26 tumor and is active in low and high immunogenic immunocompetent models MC-38 and 4 T1. **(A)** Kaplan-Meyer curve shows survival fractions in CT-26 tumor bearing mice treated at indicated doses with AZD8835 at intermittent or continuous schedule (dashed and full blue line, respectively) and intermittent and continuous treatment with PI-3065 (dashed and full green line, respectively). Data are representative of ≥2 independent experiments. (PDF 60 kb)

Additional file 2: **Table S1.** Immuno-phenotyping antibodies used for tumor flow cytometry. (DOCX 13 kb)

Additional file 3: **Figure S2.** Low dose of PI3Kα/δ inhibitor suppresses tumor T-regs independent of efficacy. **(A)** Line graph shows mean tumor volumes from BALB/c mice bearing CT-26 tumors dosed 4 days after cell implant at indicated doses. (B) Line graph shows individual tumor volumes from BALB/c mice bearing CT-26 tumors. Grey area in plot indicates continuous schedule and dashed lines indicate 2 days on/5 days off intermittent schedules at indicated doses of AZD8835 or PI-3065. (C) Scatter plots represent relative tumor T-regs cell frequencies relative to CD45+ cells. (D) Scatter plots represent tumor CD8/T-regs ratios. (PDF 86 kb)

Additional file 4: **Figure S3.** Immune phenotyping of MC-38 tumors treated with AZD8835. Scatter plot shows relative quantification of (**A)** cytotoxic CD8^+^ T-cells, **(B)** Mo-MDSCs, **(C)** DCs, **(D)** Macrophages, **(E)** G-MDSC/Neutrophil and **(F)** NK cells of treated and untreated tumors with AZD8835 (PI3Kα/δ) 50 mg/kg 2on/5off for a period of 10 days. Error bars represent mean ± SEM, statistical differences were calculated using a 1-way ANOVA with post hoc analysis. Data are representative of 2 independent experiments. Statistical significance is indicated as follows: * *p* ≤ 0.05, ** *p* ≤ 0.01, *** *p* ≤ 0.001, **** *p* ≤ 0.0001. (PDF 77 kb)

Additional file 5: **Table S2.** List of differentiated expressed genes in control and AZD8835 treated tumors at day 7 and day 14. (XLXS 59 kb)

Additional file 6: **Figure S4.** Direct target engagement in primary immune cells. (**A-B**) CD8^+^ T-cells were purified from spleens, preincubated with inhibitors AZD8835 (α/δ) AZD8186 (β/δ) and CAL101 (δ) for 1 h, then stimulated with 10 μg/mL α-CD3 and 2 μg/mL soluble α-CD28 for 25 min at 37 °C. **(A-B**) Line graph shows MFI of pAkt(Ser473) and pS6(Ser240/244), histograms show representative data. Data are representative of ≥2 independent experiments. **(C)** Cell lysates were prepared, separated by SDS-PAGE, and immunoblotted to detect pS6(Ser240/244), pNDRG-1(Thr346) and Vinculin. (PDF 307 kb)

Additional file 7: **Figure S5.** IL2 RNA and Bcl family RNA heatmap and protein levels. **(A)** Purified naïve CD8+ T-cells were rested for 3 days with AZD8835 at a maximum of 10 μM under stimulated conditions with CD3/CD28 beads. **(B)** Heatmap shows mRNA expression levels of anti-apoptotic factors on purified CD8+ T-cells treated as in A. **(C)** Histogram showing increased mean fluorescence intensity (MFI) levels of BCL2 protein in cell treated with 0.3 μM of AZD8835 and CAL-101. Data represent ≥2 experiments. (PDF 75 kb)

Additional file 8: **Table S3.** TaqMan primers panel of 96 genes used to calculate a GSVA score. (DOCX 17 kb)
