# Supplementary figures and images for: PI3Kα/δ inhibition promotes anti-tumor immunity through direct enhancement of effector CD8+ T-cell activity
Source: J Immunother Cancer. 2018 Dec 27;6:158. doi: 10.1186/s40425-018-0457-0 (PMC6307194; doi:10.1186/s40425-018-0457-0)

Figure S1.

A

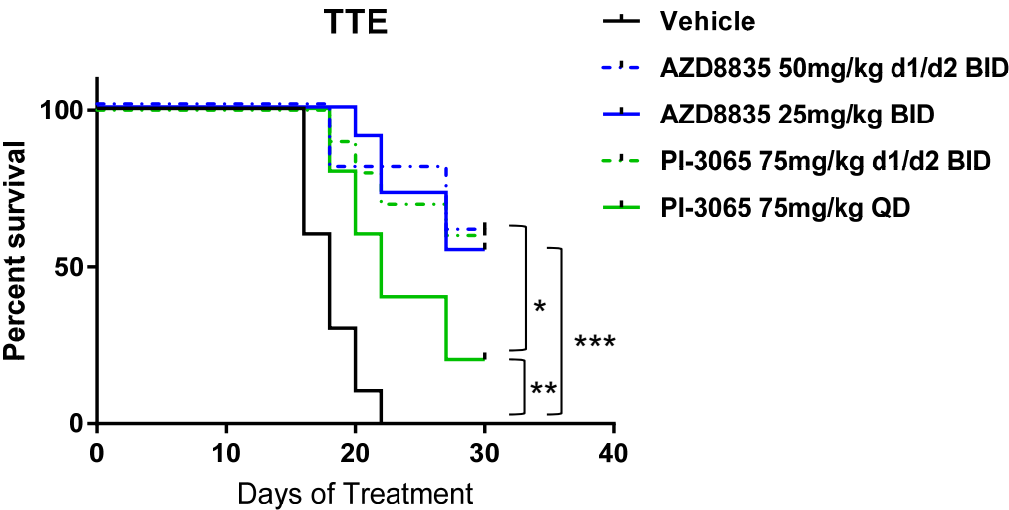

Supplement: Supplementary file 1 — Figure S1. Inhibition of PI3Kα/δ improves overall survival in CT-26 tumor and is active in low and high immunogenic immunocompetent models MC-38 and 4 T1. (A) Kaplan-Meyer curve shows survival fractions in CT-26 tumor bearing mice treated at indicated doses with AZD8835 at intermittent or continuous schedule (dashed and full blue line, respectively) and intermittent and continuous treatment with PI-3065 (dashed and full green line, respectively). Data are representative of ≥2 independent experiments. (PDF 60 kb) [file 40425_2018_457_MOESM1_ESM.pdf]

Figure S2.

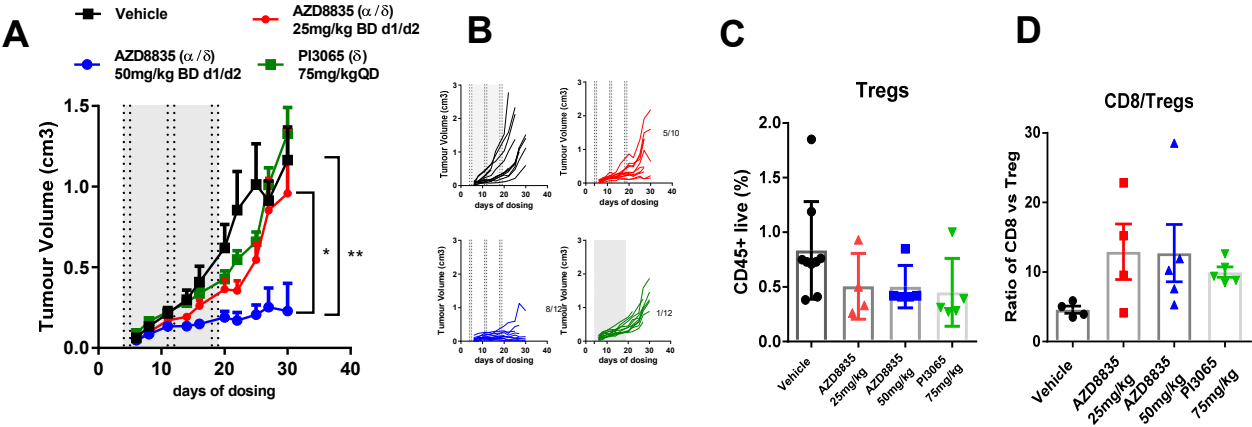

Supplement: Supplementary file 3 — Figure S2. Low dose of PI3Kα/δ inhibitor suppresses tumor T-regs independent of efficacy. (A) Line graph shows mean tumor volumes from BALB/c mice bearing CT-26 tumors dosed 4 days after cell implant at indicated doses. (B) Line graph shows individual tumor volumes from BALB/c mice bearing CT-26 tumors. Grey area in plot indicates continuous schedule and dashed lines indicate 2 days on/5 days off intermittent schedules at indicated doses of AZD8835 or PI-3065. (C) Scatter plots represent relative tumor T-regs cell frequencies relative to CD45+ cells. (D) Scatter plots represent tumor CD8/T-regs ratios. (PDF 86 kb) [file 40425_2018_457_MOESM3_ESM.pdf]

Figure S3.

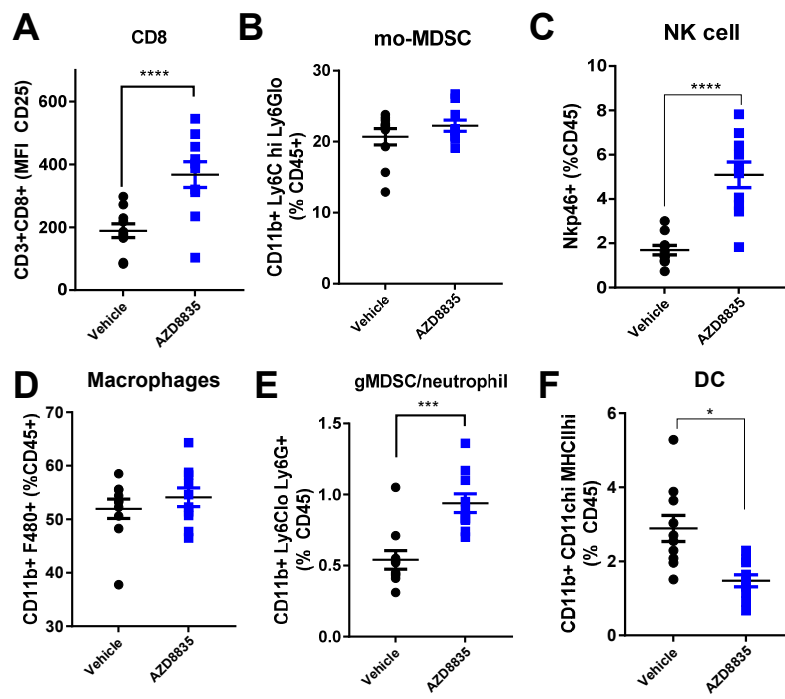

Supplement: Supplementary file 4 — Figure S3. Immune phenotyping of MC-38 tumors treated with AZD8835. Scatter plot shows relative quantification of (A) cytotoxic CD8+ T-cells, (B) Mo-MDSCs, (C) DCs, (D) Macrophages, (E) G-MDSC/Neutrophil and (F) NK cells of treated and untreated tumors with AZD8835 (PI3Kα/δ) 50 mg/kg 2on/5off for a period of 10 days. Error bars represent mean ± SEM, statistical differences were calculated using a 1-way ANOVA with post hoc analysis. Data are representative of 2 independent experiments. Statistical significance is indicated as follows: * p ≤ 0.05, ** p ≤ 0.01, *** p ≤ 0.001, **** p ≤ 0.0001. (PDF 77 kb) [file 40425_2018_457_MOESM4_ESM.pdf]

**Figure S4.**

**A**

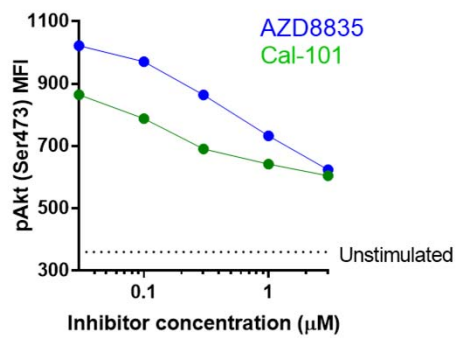

**B**

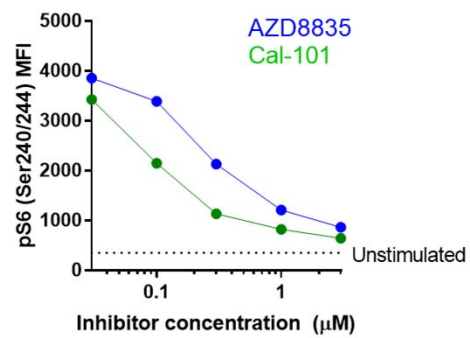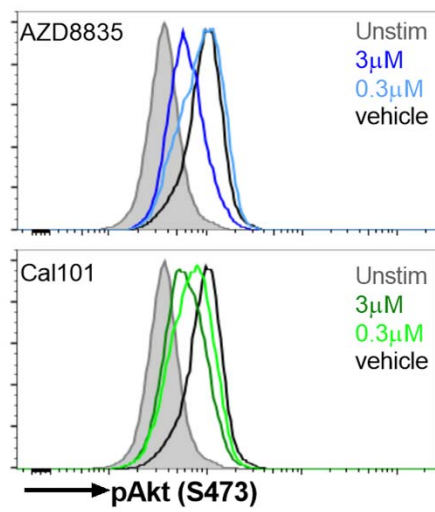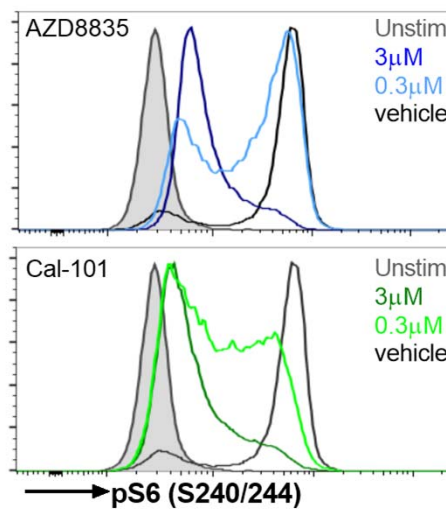

**C**

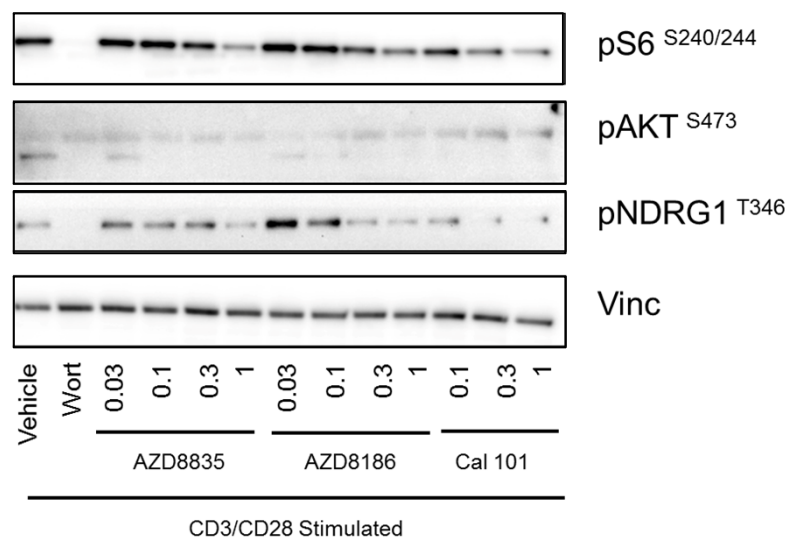

Supplement: Supplementary file 6 — Figure S4. Direct target engagement in primary immune cells. (A-B) CD8+ T-cells were purified from spleens, preincubated with inhibitors AZD8835 (α/δ) AZD8186 (β/δ) and CAL101 (δ) for 1 h, then stimulated with 10 μg/mL α-CD3 and 2 μg/mL soluble α-CD28 for 25 min at 37 °C. (A-B) Line graph shows MFI of pAkt(Ser473) and pS6(Ser240/244), histograms show representative data. Data are representative of ≥2 independent experiments. (C) Cell lysates were prepared, separated by SDS-PAGE, and immunoblotted to detect pS6(Ser240/244), pNDRG-1(Thr346) and Vinculin. (PDF 307 kb) [file 40425_2018_457_MOESM6_ESM.pdf]

Figure S5.

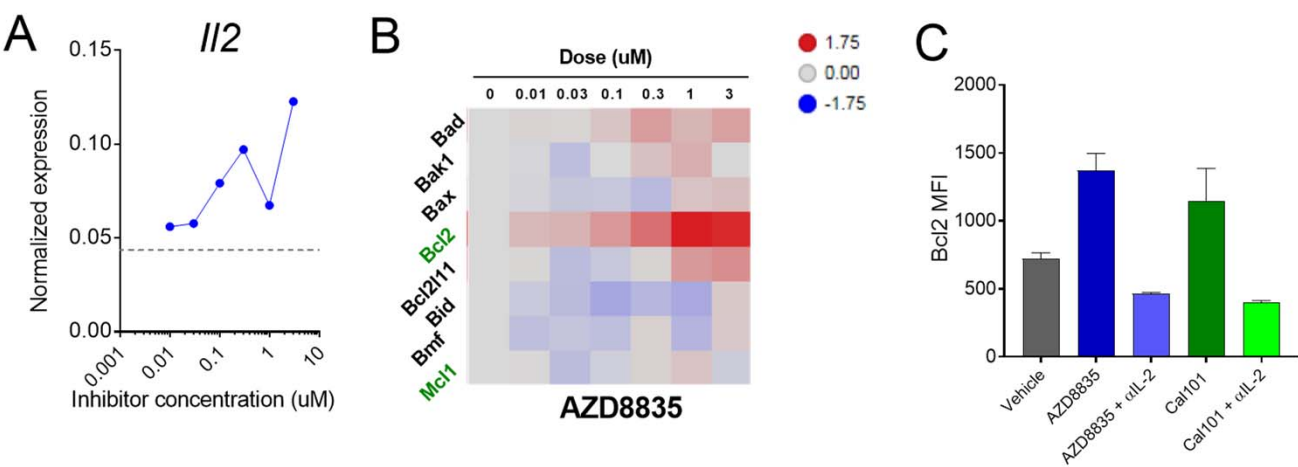

Supplement: Supplementary file 7 — Figure S5. IL2 RNA and Bcl family RNA heatmap and protein levels. (A) Purified naïve CD8+ T-cells were rested for 3 days with AZD8835 at a maximum of 10 μM under stimulated conditions with CD3/CD28 beads. (B) Heatmap shows mRNA expression levels of anti-apoptotic factors on purified CD8+ T-cells treated as in A. (C) Histogram showing increased mean fluorescence intensity (MFI) levels of BCL2 protein in cell treated with 0.3 μM of AZD8835 and CAL-101. Data represent ≥2 experiments. (PDF 75 kb) [file 40425_2018_457_MOESM7_ESM.pdf]
