# Supplementary material for: PI3Kα/δ inhibition promotes anti-tumor immunity through direct enhancement of effector CD8+ T-cell activity
Source: J Immunother Cancer. 2018 Dec 27;6:158. doi: 10.1186/s40425-018-0457-0 (PMC6307194; doi:10.1186/s40425-018-0457-0)
Supplement: Supplementary file 2 — Table S1. Immuno-phenotyping antibodies used for tumor flow cytometry. (DOCX 13 kb) [file 40425_2018_457_MOESM2_ESM.docx]

**Table S1. Immuno-phenotyping antibodies used for tumor flow cytometry.**

| **Flow cytometry panel** | **Cell Type** |
| --- | --- |
| Dead / live | Viability |
| CD45+ | hematopoietic cells |
| CD45+ CD3+ | All T-cells |
| CD45+ CD3+ CD4+ | Total CD4+ T-cell |
| CD45+ CD3+ CD4+ CD25+ FoxP3+ | T regulatory |
| CD45+ CD3+ CD4+ CD62L^+^ CD44- | Naive |
| CD45+ CD3+ CD4+CD62L^-^ CD44^+^ | Effector/Memory |
| CD45+ CD3+ CD4+ CD62L^+^CD44^+^ | Central Memory T cells |
| CD45+ CD3+ CD8+ | Cytotoxic T-cell |
| CD45+ CD3+ CD8+ CD62L^+^ CD44- | Naive |
| CD45+ CD3+ CD8+ CD62L^-^ CD44^+^ | Effector/Memory |
| CD45+ CD3+ CD8+ CD62L^+^CD44^+^ | Central Memory T-cells |
| GzmB, | Cytotoxic T cell marker |
| Ki67, | Proliferation marker |
| PD-1 | T cell Activation/exhaustion marker |
| CD45+ CD3- NKp46+ | Natural killer cells |
| CD45+ CD11b+ F4/80+ | Macrophages |
| CD45+ CD11b+ MHCII+ CD11c+ | Dendritic cells |
| CD45+ CD11b+ Ly6Chi Ly6G- | Monocytic-MDSCs |
| CD45+ CD11b+ Ly6Clo Ly6G+ | Granulocytic-MDSCs/Neutrophils |
|  |  |
|  |  |
